# Genome features and carbohydrate-active enzymes repertoire of a novel *Stenotrophomonas sepilia* Alg010 strain isolated from *Sargassum* seaweed waste

**DOI:** 10.1016/j.dib.2024.110533

**Published:** 2024-05-21

**Authors:** Bidyut R. Mohapatra

**Affiliations:** Department of Biological and Chemical Sciences, The University of the West Indies, Cave Hill Campus, Bridgetown BB11000, Barbados

**Keywords:** Biocatalyst, Biomass-degrading enzyme, Genome, Next-generation sequencing, *Sargassum*, Seaweed waste, *Stenotrophomonas*, Taxogenomics

## Abstract

This study reports the genome sequence data of a novel *Stenotrophomonas sepilia* Alg010 strain isolated from *Sargassum* seaweed waste accumulated on the coastline of Barbados. The genome sequence data was obtained via sequencing of the genomic DNA of this isolate with Illumina NextSeq2000 platform and paired-end library preparation protocol. The resulting reads were assembled with the SPAdes Genome Assembler (ver 3.15.4) and annotated with the DDBJ Fast Annotation and Submission Tool. The genome size of this novel isolate was recorded as 4,515,447 bp with a coverage of 270×, a GC content of 66.6 % and a gap ratio of 0.027 %. The lengths of the longest and the N_50_ contigs were estimated as 246,749 bp and 81,982 bp, respectively. The genome contains 2 rRNA, 66 tRNA, 2 CRISPR, 86 contigs and 4024 CDSs (coding sequences) with a coding ratio of 88.9 %. The annotation of the CDSs for COG (cluster of orthologous groups) and for subsystem features indicated that the metabolism and the amino acids and derivatives were the most dominant categories, respectively. The annotation of the genome via dbCAN3 server for carbohydrate-active genes revealed 98 genes encoding the six functional classes of carbohydrate-active enzymes. The genome sequence data is available in NCBI GenBank with the accession number BTRJ00000000.

Specifications Table

**Table:** 

Subject	Biological science
Specific subject area	Microbiology, Omics, Genomics
Data format	Raw and analyzed genome sequence
Type of data	Figures and tables
Data collection	Genome sequencing via Illumina NextSeq2000 platform
Data source location	The Alg010 strain was isolated from the *Sargassum* waste accumulated at Harrismith beach (Latitude 13.12°N and Longitude 59.42°W), Barbados
Data accessibility	Repository name: NCBI GenBank and Mendeley Data Data identification number for NCBI (accession number): BTRJ00000000 Data identification number for Mendeley (DOI): 10.17632/nfp8xcbpjt.1 Direct URL to NCBI data: https://www.ncbi.nlm.nih.gov/nuccore/BTRJ00000000 Direct URL to Mendeley data: https://data.mendeley.com/datasets/nfp8xcbpjt/1

## 1 Value of the Data

• The data presented here will be useful in comparative genomics and phylogenomics to assess the evolutionary relatedness and metabolic traits of *Stenotrophomonas* species.
• This data is important in assessing the biotechnological potential of the *Stenotrophomonas* species as biocatalysts via identification of protein-coding genes, including the carbohydrate-active enzymes.
• The genome sequence data is valuable in tailoring the genes encoding the carbohydrate-active enzymes with rational and/or combinatorial approaches for sustainable biotransformation of lignocellulosic and seaweed waste into biofuels and value-added products for potential industrial applications.

## 2 Background

The depletion of easily accessible crude oil reserves and its adverse impact on climate change via production of greenhouse gases necessitates development of green technologies for the sustainable production of renewable energies. Hence, biorefining has emerged as an alternative to petroleum refining for the environmentally friendly biotechnological processing of renewable feedstock, especially biological waste (lignocellulosic and seaweed biomass) into biofuels and value-added chemicals [1,2].

The major hurdle in biosaccharification of the polysaccharide content of lignocellulosic and seaweed waste is the requirement of expensive carbohydrate-active enzyme (CAZyme) cocktails [3]. The CAZymes are classified into six classes, glycoside hydrolases (GH), glycosyl transferases (GT), carbohydrate esterases (CE), polysaccharide lyases (PL), auxiliary activities (AA) and carbohydrate-binding modules (CBM) [4]. Research has already been undertaken to screen out potent biomass-degrading microorganisms from various environmental niches for applications as biocatalysts [5]. The advantages of the application of biocatalysts in the processing of biological waste are the operation of bioprocesses at ambient temperatures and pressures and the reduced generation of secondary pollution [6].

The recent advent of culturomics and whole-genome sequencing have fuelled on assessment of the phylogeny and metabolic potential of novel biomass-degrading microbial isolates [7]. The genomic approach is instrumental in identifying the prevalence of the CAZymes repertoire, especially the polysaccharide utilization loci (PUL). PUL is a cluster of physically linked genes that assists in saccharification of complex carbohydrates via encoding CAZymes and other protein ensembles [8].

The species of the genus *Stenotrophomonas* are of biotechnological importance in agriculture, biocontrol and bioremediation with the exception of the multidrug resistant opportunistic pathogen *Stenotrophomonas maltophila* [9]. In view of the potential biotechnological importance of the *Stenotrophomonas* genus, it is essential to assess the metabolic traits at the molecular level via whole genome sequencing. The current study describes the genome features and CAZymes repertoire of a novel *Stenotrophomonas sepilia* Alg010 strain isolated from the decomposed *Sargassum* biomass (*Sargassum* waste) inundating Barbados’ coast.

## 3 Data Description

**Fig. 1:**
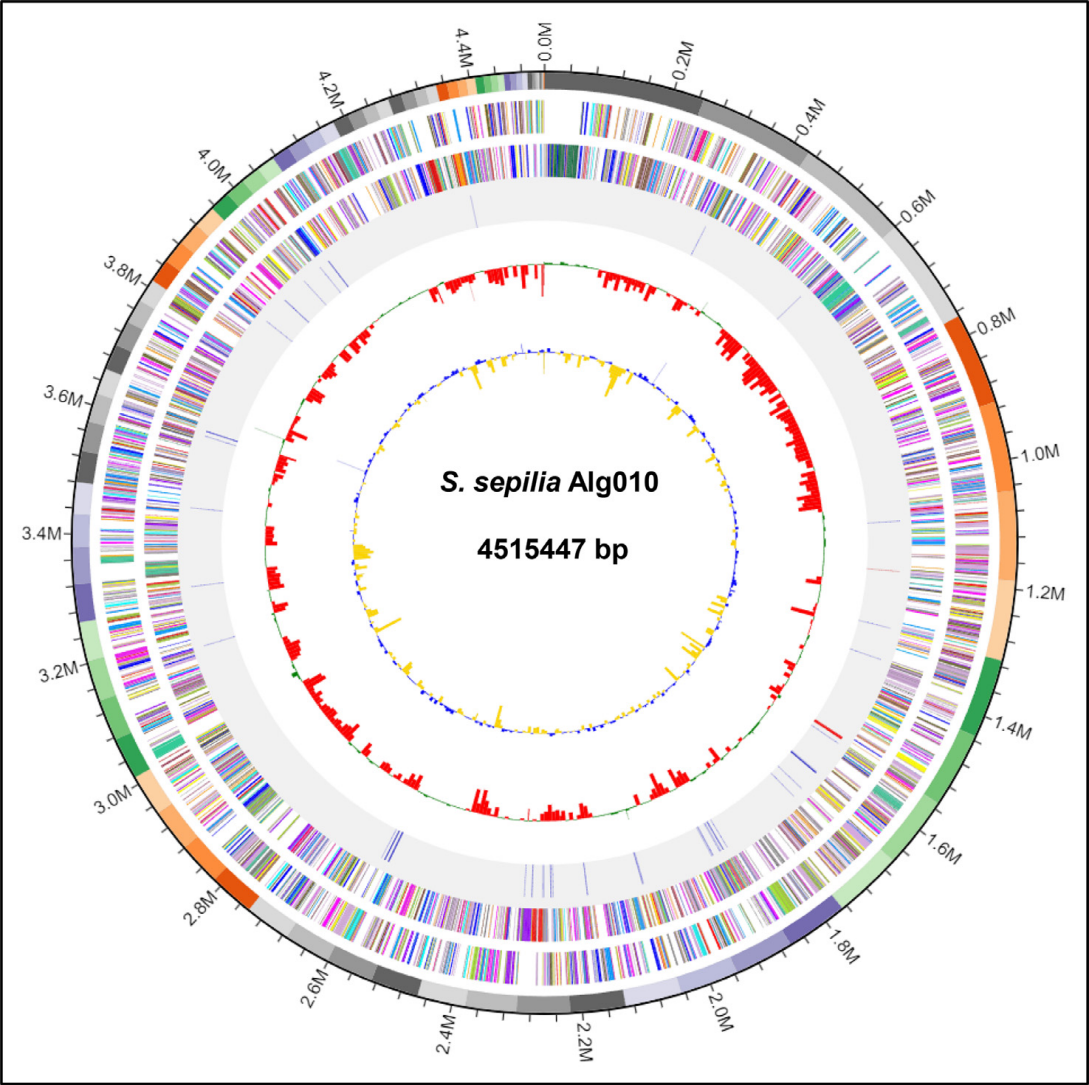
A circular genome map of *Stenotrophomonas sepilia* Alg010. From outside to inside, ring 1 denotes the 86 assembled contigs; rings 2 and 3 denote the predicted CDSs in forward and reverse strands, respectively. Each CDS is marked with a color based on its COG category (https://help.ezbiocloud.net/cog-colors/); ring 4 denotes rRNA and tRNA; ring 5 denotes GC skew; ring 6 denotes GC ratio.

**Fig. 2:**
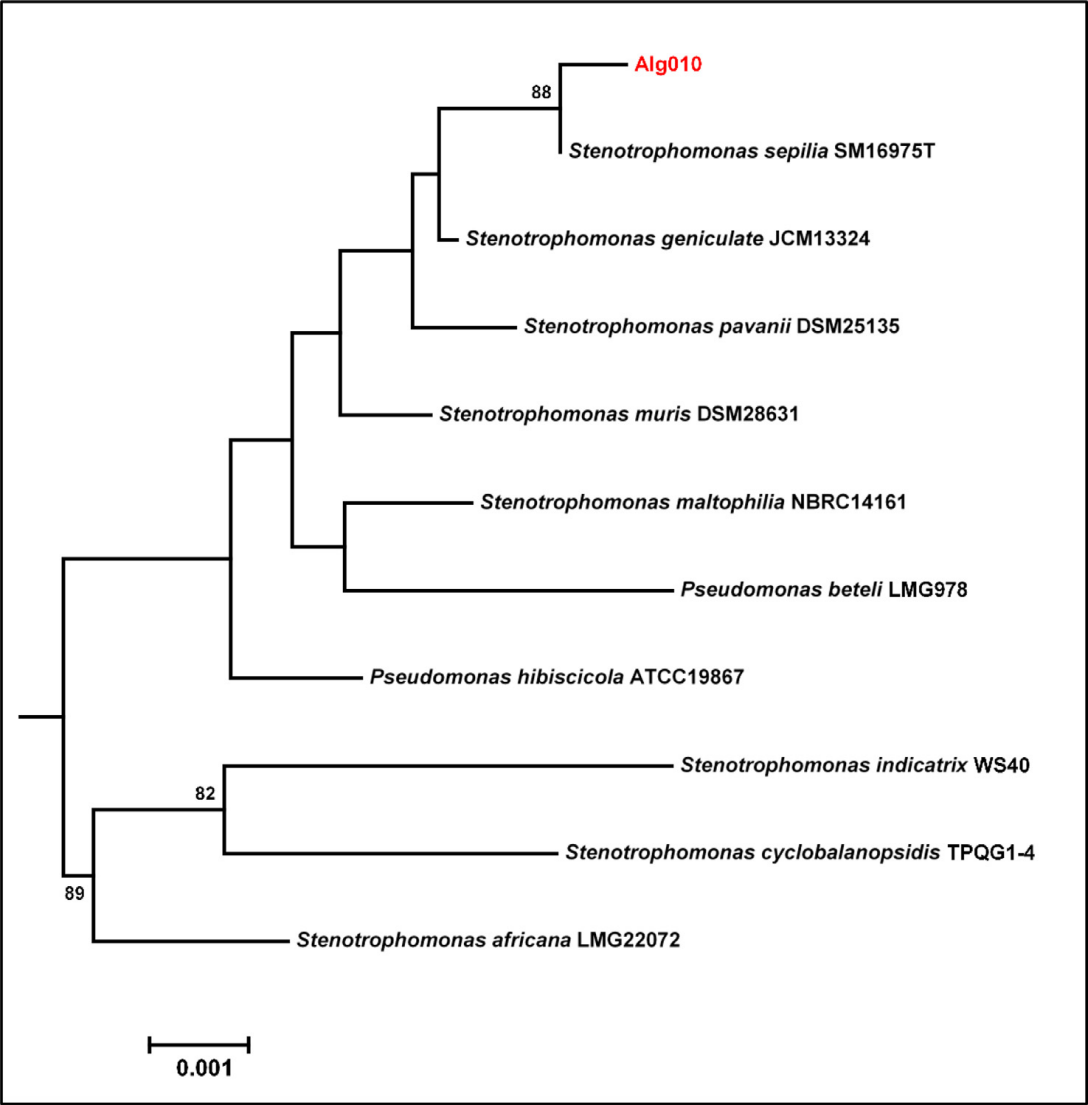
Phylogeny of the novel *Stenotrophomonas sepilia* Alg010 strain based on 16S rRNA gene sequence data. The tree was generated with FastME 2.1.6.1 [16] from Genome Blast Distance Phylogeny (GBDP) distances estimated from 16S rDNA gene sequences. The numbers above the branches denote GBDP pseudo-bootstrap support values > 60 % from 100 replications, with an average branch support of 56.4 %. The δ statistics of the tree were estimated as 0.35–0.5.

**Fig. 3:**
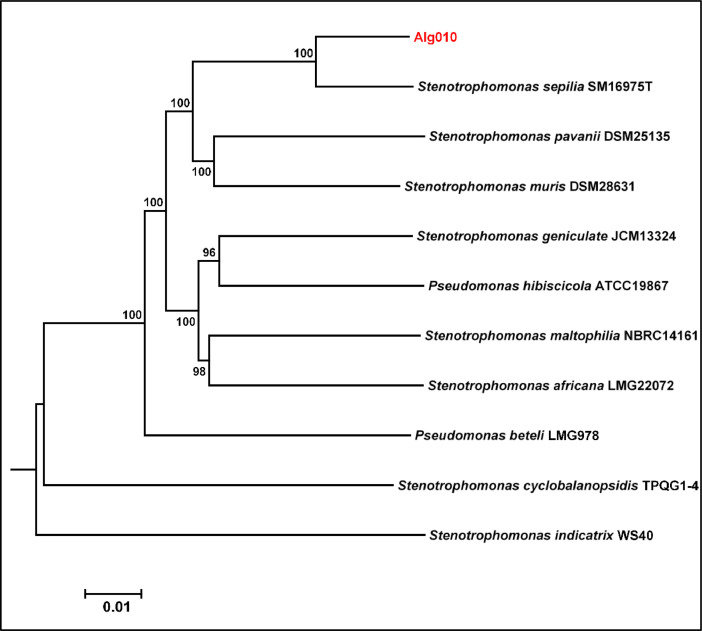
Phylogenomic tree of the novel *Stenotrophomonas sepilia* Alg010 strain based on genome sequence data. The tree was generated with FastME 2.1.6.1 [16] from Genome Blast Distance Phylogeny (GBDP) distances estimated from genome sequences. The numbers above the branches denote GBDP pseudo-bootstrap support values >60 % from 100 replications, with an average branch support of 99.3 %. The δ statistics of the tree were estimated as 0.203–0.348.

The whole genome sequence of Alg010 generated 4515,447 bp of nucleotides with a coverage of 270X. The assembly of the genome via SPAdes (ver 3.15.4) yielded 86 contigs with the values obtained for the longest and N_50_ contigs being 246,749 bp and 81,982 bp, respectively. The GC content of the genome was recorded as 66.6 %. The completeness check of the genome by CheckM [10] resulted in values of 99.6 % completeness and 1.22 % contamination. Subsequent annotation via DFAST revealed the existence of 2 rRNA, 66 tRNA, 2 CRISPR and 4024 CDSs (coding sequences) with a coverage of 88.9 % (Table 1). The circular genome map of Alg010 displaying the contigs, GC content and GC skewness, is shown in Fig. 1. The phylogenomic analysis of Alg010 genome via Type (Strain) Genome Server (TYGS) unveiled its affiliation as *Stenotrophomonas sepilia* (GCA_003244875) with a digital DNA-DNA hybridization (dDDH) value of 71.2 % and an average nucleotide identity (ANI) value of 96.9 %. The evolutionary relationships of the genome and the 16S rRNA gene of the novel *Stenotrophomonas sepilia* Alg010 strain with other closely related species are displayed in Figs. 2 and 3, respectively. This novel strain was predicted to be a nonpathogenic bacterium via screening of its genome in PathogenFinder 1.1.

**Table 1:** Genome features of the *Stenotrophomonas sepilia* Alg010 strain.

Feature	*S. sepilia* Alg010
Genome size	4515,447 bp
Genome coverage	270X
Number of contigs	86
GC content	66.6 %
Longest contig	246,749 bp
N50	81,982 bp
Gap ratio	0.027 %
CDS	4024
rRNA	2
tRNA	66
CRISPR	2
Coding ratio	88.9 %

**Fig. 4:**
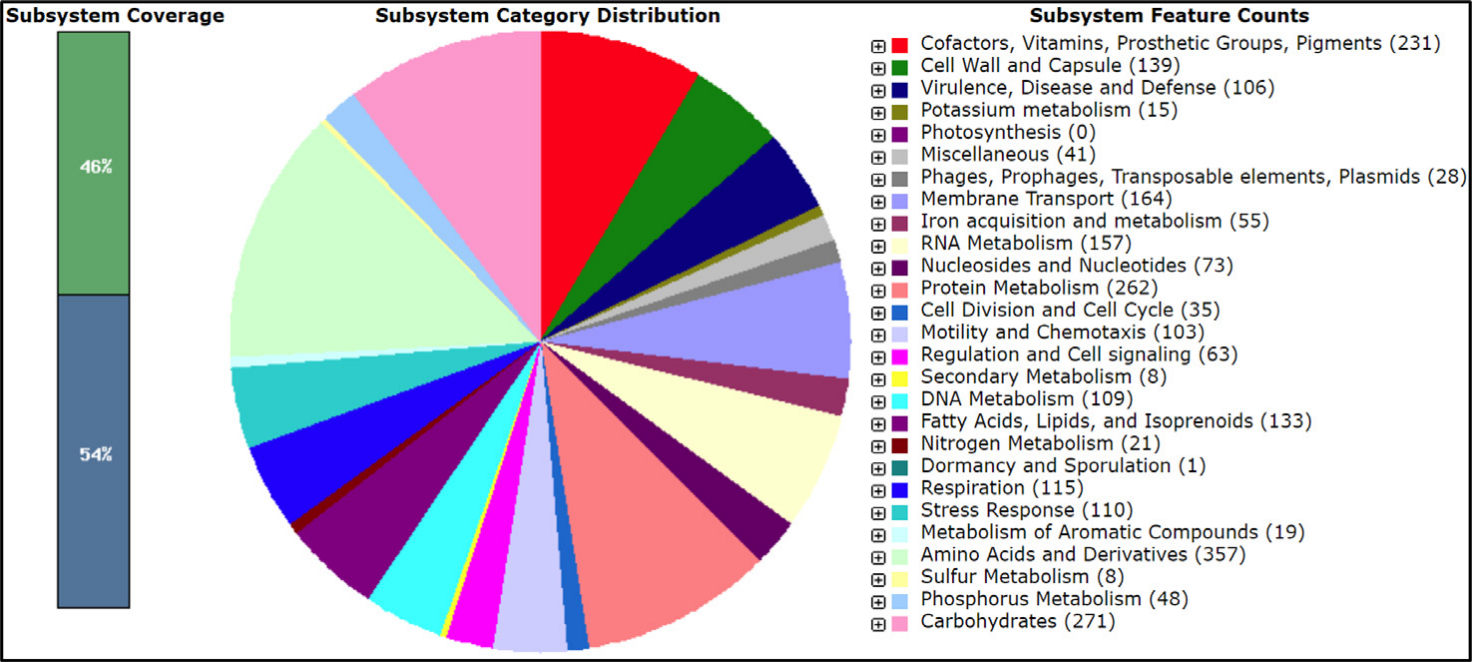
RAST annotation with subsystem category distribution of the *Stenotrophomonas sepilia* Alg010 genome. Subsystem coverage and subsystem feature counts are specified in the legends.

The annotation of the CDSs using COG (Clusters of Orthologous Genes) database revealed that the majority of the genes (68 %) were assigned with a COG functional category (Fig. 1). For the COG functional class, the metabolism (29.6 %) was found to be dominant, followed by the cellular process and signaling (24.1 %), and the information storage and processing (14.3 %). These genes were clustered into 22 COG categories (Table S1 in Mendeley Data). The functional annotation of the Alg010 genome via RAST predicted 3989 CDS with 2107 features classified into 447 subsystems (Fig. 4). The three most represented subsystem categories in Alg010 genome were recorded as the amino acids and derivatives (13.4 %), the carbohydrates (10.1 %) and the cofactors, vitamins, prosthetic groups and pigments (9.81 %).

The evaluation of the diversity of CAZyme genes in the Alg010 genome indicated the presence of 98 genes, which were classified into six classes of glycan synthesis, modification and degradation. The highest CAZyme class detected in Alg010 genome was GH (38 genes), followed by GT (36 genes), CE (12 genes), AA (6 genes), PL (1 gene) and CBM (5 genes) (Table 2). The enzymes of class GH catalyze the hydrolysis and/or rearrangement of glycosidic bonds. The four abundant families of the GH class were recorded as GH23 (7 genes), GH3 (5 genes), GH43 (3 genes) and GH92 (3 genes). The predicted enzymes of GH23, GH3, GH43 and GH92 catalyze the degradation of carbohydrates by chitinase, glucosidase/glucanase/xylosidase, arabinofuranosidase and mannosidase, respectively. The enzymes of the GT class catalyze the formation of the glycosidic linkage to produce a glycoside. The GT2 (15 genes), GT4 (6 genes) and GT51 (4 genes) were the three most dominant families of the GT class present in the genome of Alg010. These three families of GT contain glycosyl synthase and glycosyl transferase. The enzymes of the CE class catalyze the hydrolysis of the carbohydrate esters of polysaccharides. The three abundant CE families, CE4 (4 genes), CE9 (2 genes) and CE14 (2 genes) were reported as catalyzing the hydrolysis of acetylated xylan and chitin, acetylated glucosamine and diacetyl chitobiose, respectively. The enzymes of the AA class contain the redox enzymes that act in conjunction with the CAZymes in the degradation of carbohydrates. The dominant family of the AA class recorded in the Alg010 genome was AA10 (3 genes). AA10 is capable of oxidative cleavage of xylan, chitin and cellulose. The PL class, which catalyzes the non-hydrolytic cleavage of glycosidic bonds, contain only one gene of the family PL5. CBM are non-catalytic modules of CAZymes and catalyze the reaction by adhesion to carbohydrates. The dominant CBM was recorded as CBM50 (3 genes). CBM50 binds to the enzymes responsible for the degradation of chitin or peptidoglycan.

**Table 2:** Carbohydrate-active enzymes (CAZymes) in the *Stenotrophomonas sepilia* Alg010 genome.

CAZyme class	CAZyme family (number of genes)
Auxiliary activities (AA)	AA1 (1), AA3 (1), AA6(1), AA10(3)
Carbohydrate-binding modules (CBM)	CBM32 (2), CBM50 (3)
Carbohydrate esterases (CE)	CE1 (1), CE4 (4), CE9 (2), CE11 (1), CE12 (1), CE 14(2), CE20 (1)
Glycoside hydrolases (GH)	GH2 (1), GH3 (5), GH13 (2), GH15 (1), GH18 (2), GH19 (2), GH20 (1), GH23 (7), GH24 (1), GH31 (1), GH43 (3), GH67 (1), GH73 (1), GH92 (3), GH97 (1), GH102 (1), GH103 (2), GH108 (2), GH144 (1)
Glycosyl transferases (GT)	GT (1), GT2 (15), GT4 (6), GT9 (1), GT19 (1), GT20 (1), GT28 (1), GT30 (1), GT41 (1), GT51 (4), GT83 (3), GT105 (1)
Polysaccharide lyases (PL)	PL5 (1)

The detection of polysaccharide utilization loci (PUL) in Alg010 genome via BLASTX search against PUL proteins database of dbCAN-PUL indicated the presence of 41 CAZymes, 9 STPs (signal transduction protein), 88 TCs (transported classification) and 20 TFs (transcription factor). The PUL with the highest hit to the query sequences was PUL0169 (15 hits) (Table S2 in Mendeley Data). The PUL0169 is reported to be capable of saccharification of arabinogalactan. This genome annotation data is valuable in assessing the biocatalytic potential of the *Stenotrophomonas sepilia* Alg010 strain for sustainable bioprocessing of lignocellulosic and seaweed waste.

## 4 Experimental Design, Materials and Methods

### 4.1 Isolation of microorganisms

*Stenotrophomonas* sp. Alg010 used in this study was isolated from the decomposed *Sargassum* biomass accumulated at Harrismith beach (Latitude 13.12°N and Longitude 59.42°W), Barbados, via tenfold serial dilutions in sterile seawater and spread plating on mineral agar (0.02 % MgSO_4_, 0.002 % CaCl_2_, 0.1 % KH_2_PO_4_, 0.1 % K_2_HPO_4_, 2 % NaCl, 0.1 % NH_4_NO_3_ and 1.5 % agar; pH 7.5) supplemented with 0.5 % (w/v) sodium alginate (Sigma Chemicals, Milwaukee, WI) as described before [11]. The decomposed seaweed biomass was composed of two holopelagic species of *Sargassum, S. fluitans* and *S. natans*.

### 4.2 Genome sequencing and annotation

The genomic DNA was extracted using InstaGene™ matrix (Bio-Rad Laboratories, Canada) according to the manufacturer's instruction. The extracted DNA was purified using ethanol precipitation [12] and then resuspended in elution buffer (IBI Scientific, Peosta, IA). The isolated genomic DNA of *Stenotrophomonas* sp. Alg010 was sequenced via Illumina NextSeq2000 platform with 2 × 150 bp paired-end reads and assembled with SPAdes Genome Assembler (ver. 3.15.4). The assembled genome of Alg010 was annotated using the DDBJ Fast Annotation and Submission Tool (DFAST) for estimating the average nucleotide identity (ANI) value and identifying the CDSs (coding sequences), tRNA genes, rRNA and CRISPR [13]. Additional genome annotation was performed via the RAST for mapping of genes to subsystems [14] and the eggNOG mapper (ver. 4.1) [15] for determining the clusters of orthologous groups (COG) of proteins. The circular map of the genome was generated via the EzBioCloud pipeline (https://eztaxon-e.ezbiocloud.net/).

### 4.3 Phylogeny of Alg010

The phylogeny of Alg010 was assessed in the Type (Strain) Genome Server (TYGS) using the whole-genome sequence data and Genome BLAST Distance Phylogeny (GBDP) approach [16]. Two strategies were used for determining the identity of the Alg010 isolate. Firstly, the genome of Alg010 was compared with all the type strain genomes available in TYGS database using the MASH algorithm to determine intergenomic relatedness. Secondly, the curated 16S rRNA gene sequence of Alg010, from the whole-genome sequence, was compared with the 16S rRNA gene sequences of the type strains available in the TYGS database. Digital DNA-DNA hybridization (dDDH) values and confidence intervals were estimated in TYGS for the ten closely related type strains of Alg010.

### 4.4 Pathogenicity detection

The pathogenic status of the Alg010 isolate was determined from its whole-genome sequence using PathogenFinder (ver. 1.1) [17].

### 4.5 CAZyme and PUL identification

The Alg010 genome was further annotated with reference to the CAZymes and their substrate specificity via the dbCAN3 pipeline [18]. The dbCAN3 integrates three state-of-the-art tools/databases for automated CAZyme annotation: HMMER search for CAZyme family annotation vs. dbCAN CAZyme domain HMM database; DIAMOND search for BLAST hits in the CAZy database; and HMMER search for CAZyme subfamily annotation vs. dbCAN-substrate HMM database of CAZyme subfamilies. The annotation results of the CAZymes are reported if two of the specified tools identified them [18]. The occurrence of PULs in the genome of Alg010 was assessed via BLASTX search of dbCAN-PUL proteins repository in the dbCAN-PUL pipeline [19].

## Limitations

None

## Ethics Statement

The author has read and follow the ethical requirements for publication in Data in Brief and confirming that the current work does not involve human subjects, animal experiments, or any data collected from social media platforms.

## CRediT authorship contribution statement

**Bidyut R. Mohapatra:** Conceptualization, Methodology, Formal analysis, Writing – original draft, Writing – review & editing.

## Data Availability

Stenotrophomonas sepilia strain Alg010, whole genome shotgun sequencing project (Original data) (NCBI GenBank)
